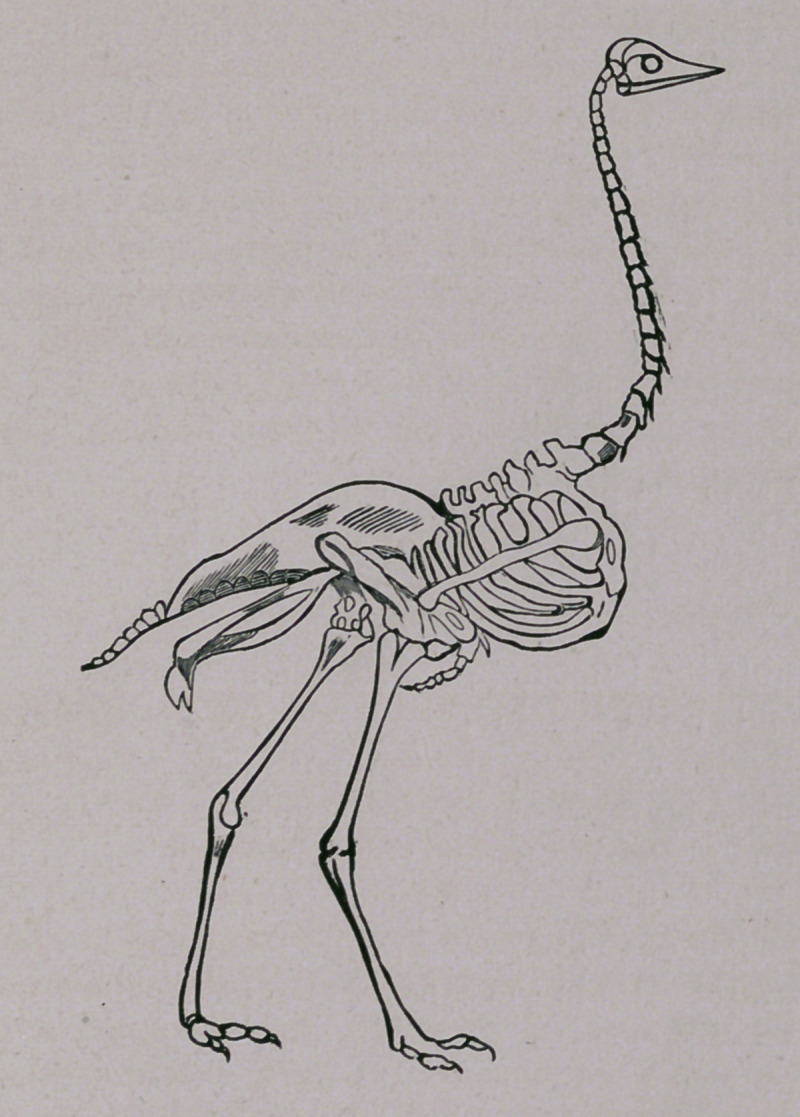# Some Diseases of the Ostrich

**Published:** 1884-01

**Authors:** W. A. Conklin

**Affiliations:** Director of the Central Park Menagerie


### Art. II.—SOME DISEASES OF THE OSTRICH.

BY W. A. CONKLIN, D.V.S.,
*Director of the Central Park Menagerie.*

The probable establishment in this country of ostrich farms suggested by the recent importation of a large number of these birds for the purpose of breeding, and the general interest awakened in this novel branch of industry would seem to make their diseases a fitting subject of attention for veterinarians.

A few words relating to the anatomy and physiology of the ostrich will not be amiss.

The skeleton of the ostrich is marked by a few peculiarities which distinguishes it from that of other birds. The wings are rudimentary and not adapted for flight, consequently we find that the clavicles do not come in contact inferiorily, although they reach the sternum, and that the radius and ulna are but one-third the length of the humerus. The inner metatarsal bone terminates in a point near the base of the great trochlea, the outer trochlea being comparatively small and short. Birds, as a rule, have four toes. The ostrich proper has but two, the third and fourth being those retained, and although the latter is much the smaller and shorter toe, it still holds its superior number of joints. The other members of the ostrich family have three toes. The sternum, which has no keel, is merely a convex, bony shield, but is covered with a callous pad, or cushion, having a hard, rough surface, denuded of feathers, on which the bird leans when at rest.

The muscular mechanism of the leg is as interesting a study as may be found in the animal kingdom.

Dr. Houghton thus describes it: "The leg of the ostrich is to be regarded as a long rod bent at four distinct points, which attains its greatest amount of shortening or bending at the moment the foot touches the ground, and which is suddenly straightened or elongated by the simultaneous contraction of all the muscles. The effect of the sudden elongation of the leg is to throw the whole body of the bird forward, as if from a catapult, from the point of support of the foot, and while the body of the animal is thus projected through the air the antagonist muscles that flex the several joints come into play, and are assisted in their action by some very remarkable contrivances in the heel joint."

The ostrich has the most complicated digestive apparatus in the whole class of birds.

Owen describes it as follows: "The duodenal fold is about a foot in length and the returning part makes a bend upon itself before it reaches the pylorus, the intestine then turns down again behind the duodenal folds and gradually acquires a wider mesentery. The ileum after a few folds ascends toward the left side, accompanied by two long cœca and becomes again connected with the posterior part of the duodenal mesentery, beyond which the cœca enter the intestine behind the root of the mesentery, and the large intestine commences. This part differs from the rectum in other birds in its great extent, being nearly double the length of the small intestines and being disposed in folds upon a wide mesentery. It terminates by an oblique valvular aperture in a large urinary receptacle. The cœca are wide, upwards of two feet each in length, and their secreting and absorbing parietes are further increased by being produced into a spiral valve, analagous to that which exists in the long cœcum of the hare and rabbit."

In its natural state in the sandy deserts the ostrich procures its food supplies, which are not of a very digestible nature, at long intervals, hence the necessity of a digestive apparatus capable of extracting the entire nutritious matter the food contains. This bird indeed is proverbial for its remarkable digestion, it being well known that nails, pieces of wood, etc., are readily dissolved in the bird's stomach, yet it would appear that one of the principal causes of its disease is imperfect digestion, the result most frequently of voraciousness. It is an established fact that the more complicated an organ is the more readily is it deranged, and in practice we find that the ostrich suffers more than any other bird from diseases of the digestive organs.

The senses of sight and smell in this bird are very acute.

The yield and quality of the feathers appear to be directly in proportion to the health and vigor.

The grazing ground best suited for the ostrich is that in which the soil and plants are rich in alkalies. When this is not the case art should supply the needful element.

Phosphate of lime in the form of bone dust is an excellent food.

Considering the disorders to which these birds are susceptible in the order of their frequency we will take up first, as might be expected.

*Indigestion.*—This is generally caused by gorging, or overfeeding. It is often accompanied by constipation or stop-sickness, in which case the prognosis is unfavorable. This condition will be ascertained by examining the neck and ventriculus, which parts are found to be of unusual hardness.

In the treatment the first thing to be done is to produce a passage from the bowels. For this purpose a dose of from eight to ten ounces of castor oil in a capsule, or one pound of Epsom salts will prove effectual for a full grown bird. A simple remedy can be at once given. It consists of Indian meal moistened with oil, a small quantity of salt being added. Rock salt broken in small pieces and mixed with pebbles should be placed within the bird's reach.

When constipation is obstinate and resists simple treatment the hardened feces may be removed by inserting the hand into the rectum. In the meantime enemas of soap and water are to be used, care being taken not to use force and to keep the syringe pointing in an upward direction. The bird should be fed on soft, plain food for some time after.

As a preventive measure nothing is better than regularity of feeding and limiting the number of meals to two daily.

The enclosure of the young bird should be inspected with great care, and if anything unusual is noted, either in the walk or general condition of the birds, they should be removed immediately to a dry, warm stable.

Care should be taken in the selection of the food for the birds. They thrive well on barley, fresh cabbage and the leaves of the cactus, or barbary fig, cut fine. Each bird requires about three pounds averdupois of barley a day with green food, according to circumstances. Water should be supplied freely, as although the ostrich can endure long periods of fast, being nourished at those time by its own fat, it cannot so well endure thirst.

*Worms.*—The domesticated ostrich suffers very often from tape worm. Formerly these parasites caused great mortality among the birds, but now effectual remedies are found for this particular worm, and the disease is no longer much dreaded.

The worms generally appear in chicks about four months old. They are located in the small intestines, consuming all the best part of the food, so that the bird becomes greatly emaciated unless an extra amount of food is given it.

The best vermifuges are turpentine and male fern in the following doses: Half an ounce of turpentine, or one and a half drachms of male fern to a chick of four months. increasing the dose according to age in the same proportion.

Care should be taken to put all liquid medicines in capsules and pass well down the gullet so that no portion of it shall go into the wind pipe. A good, cheap capsule, and readily obtained, can be made from the lining of a sausage.

Another species of worm inhabiting the stomach of the ostrich is the "Strongylus Douglassii," so named from Mr. Douglass, who discovered this worm a few years ago. It is about one-fourth of an inch long, of white color, and is found in the mucous membrane, causing severe inflammation of the stomach.

The presence of these worms in the ostrich has been caused by the abscence of alkalies in the food used. The appearance of the bird when affected with this disease is very much altered. It is found to be reduced in flesh, with ruffled feathers, drowsiness, etc. The bird rushes greedily for the food and after taking a few mouthfuls turns away, evidently in great pain as the food enters the stomach, and after repeating the attempt a few times finally gives it up altogether.

As regards the treatment we must confine ourselves to those remedies best calculated to keep up the general strength, as we possess no vermifuge against this worm.

The bones of the ostrich often become diseased, owing to poor or insufficient diet, and as a result extreme weakness supervenes. Bread pills, each containg five grains of citrate of iron and quinine, given twice a day, will speedily effect a cure.

The ostriches in this country under my care have been lately attacked by diphtheria, the disease ending fatally in every case. They have suffered also from convulsions due, apparently, to indigestion.

*Injuries.*—Wounds, as a rule, get well without special treatment. When the bones of the leg are fractured the bird should be killed, as an attempt to procure union would entail much expense and trouble. Dislocations, if seen soon after the accident, can be readily reduced and will quickly heal if quiet be maintained for a few days. Foreign bodies lodging in the œsophagus may either be extracted through the mouth or pushed down into the stomach, whence, aided by a cathartic, they obtain exit *per anum.* When these means fail the object may be removed through an incision made in the throat at the point of lodgement, care being taken that the trachea be not injured during the operation. Draw the skin to one side before making the incision, so that the wound in the œsophagus will not be in line with that of the integrement and so it will be well protected from iujurious external influences. In injuries of the legs produced during violent struggle or while being entangled in a wire fence, rest and applications of carbolised oil will generally bring about a cure. Injuries of the head, even very slight ones, generally end fatally.

## Figures and Tables

**Figure f1:**